# Harnessing bacterial strain from rhizosphere to develop indigenous PGPR consortium for enhancing lobia (*Vigna unguiculata*) production

**DOI:** 10.1016/j.heliyon.2023.e13804

**Published:** 2023-02-17

**Authors:** Jay Prakash Verma, Durgesh Kumar Jaiswal, Anand Kumar Gaurav, Arpan Mukherjee, Ram Krishna, Arthur Prudêncio de Araujo Pereira

**Affiliations:** a Plant Microbes Interaction Lab, Institute of Environment and Sustainable Development, Banaras Hindu, University, Varanasi, 221055, Uttar Pradesh, India; b Soil Microbiology Laboratory, Soil Science Department, Federal University of Ceará, Fortaleza, Ceará, Brazil

**Keywords:** Rhizosphere soil, Soil microbes, Plant growth-promoting rhizobacteria (PGPR), Plant growth promoting activities, Lobia (*Vigna unguiculata*)

## Abstract

The rhizosphere microbes play a key role in plant nutrition and health. However, the interaction of beneficial microbes and *Vigna unguiculata* (lobia) production remains poorly understood. Thus, we aimed to isolate and characterize the soil microbes from the rhizosphere and develop novel microbial consortia for enhancing lobia production. Fifty bacterial strains were isolated from the rhizosphere soil samples of lobia. Finally, five effective strains (e.g., *Pseudomonas* sp. IESDJP-V1 and *Pseudomonas* sp. IESDJP-V2, *Serratia marcescens* IESDJP-V3, *Bacillus cereus* IESDJP-V4, *Ochrobactrum* sp. IESDJP-V5) were identified and molecularly characterized by 16 S rDNA gene amplification. All selected strains showed positive plant growth promoting (PGP) properties in broth culture. Based on morphological, biochemical, and plant growth promoting activities, five effective isolated strains and two collected strains (*Azospirillum brasilense* MTCC-4037 and *Paenibacillus polymyxa* BHUPSB17) were selected. The pot trials were conducted with seed inoculations of lobia *(Vigna unguiculata)* var. Kashi Kanchan with thirty treatments and three replications. The treatment combination T3 (*Pseudomonas* sp. IESDJP-V2), T14 (*Pseudomonas* sp. IESDJP-V2 + *A. brasilense*), T26 (*Pseudomonas* sp. IESDJP-V1+ *B. cereus* IESDJP-V4 + *P. polymyxa*) and T27 (IESDJP-V1+ IESDJP-V5+ *A. brasilense*) were recorded for enhancing plant growth attributes, yield, nutritional content like protein, total sugar, flavonoid and soil properties as compared to control and others. The effective treatments T3 (*Pseudomonas* sp.), T14 (*Pseudomonas* sp. IESDJP-V2 + *A. brasilense*), T26 (*Pseudomonas* sp. IESDJP-V1+ *B. cereus* IESDJP-V4 + *P. polymyxa*) and T27 (IESDJP-V1+ IESDJP-V5+ *A. brasilense*) recorded as potential PGPR consortium for lobia production. The treatment of single (*Pseudomonas* sp.), duel (IESDJP-V2 + *A. brasilense*) and triple combination (IESDJP-V1+ IESDJP-V4 + *P. polymyxa*) and (IESDJP-V1+ IESDJP-V5+ *A. brasilense*) can be further used for developing effective indigenous consortium for lobia production under sustainable farming practices. These PGPR bio-inoculant will be cost-effective, environment-friendly and socially acceptable.

## 1 Introduction

World's population is continuously increasing and to fulfil the basic need (food, fiber and energy) of the population, we have to improve crop productivity more and more in near future [1,2]. The green revolution that affected the crop production by hampering the soil properties, mainly its fertility and, consequently, microbial communities. The extensive use of mineral fertilizers and pesticides causes environmental pollution and health issue for the farmers. Moreover, imbalance use of agro-chemical and un-sustainable farming practices are the major threats to poor productivity due to loss of soil fertility and health and environmental degradation [[3], [4], [5], [6], [7]]. Hence, there is a crucial prerequisite to finding out alternative approaches which can boost crop productivity by delivering environment friendly, socially acceptable, and economically viable technology to sustain long-term ecological balance in different agro-ecosystem. The alternatives solution is the application of different organic farming practices like plant growth-promoting rhizobacteria, biofertilizers, biopesticides, microbial consortium of plant growth promoter, integrated nutrient management, crop rotation, mixed cropping, vermicomposting, biofortification of organic residues, bio-composting, etc. Nowadays, plant growth-promoting rhizobacteria (PGPR) is an important concept of microbial groups which promote plant growth attributes by direct and indirect mechanisms [[8], [9], [10]].

The application of microbial consortium or inoculum in single, dual, triple, tetra, penta and hexa, and more combinations are widely used for increasing sustainable agricultural productivity as well as enhancing soil fertility and health [6,8]. PGPR are free-living soil microorganisms which have ability to easy colonize in the rhizosphere and promote plant growth attributes due to application of seed, seedling, soil, and foliar methods [6,11,12].

Cowpea/lobia (*Vigna unguiculata* L.) is an important seed legume crop that is broadly used as green vegetables in India and other countries of World [13]. It is not only used for human consumption but also used as fodder, and green manure crops for increasing nitrogen and organic matter in soils. Low productivity of lobia is one of the important reason non-effective microbial inoculants as well as poor soil fertility and health, phytopathogens and seed quality. Hence, the application of PGPR improves nutrient availability for lobia productivity is an important approach. da Braulio [14] isolated dizotrophic bacterial strains INPA 03–11B, UFLA 03–84, UFRB FA34C2-2 and did seed inoculation with *Vigna unguiculata* (L.) for enhancing plant tolerance, productivity and nutrient content (Nitrogen and Phosphorus). Costa and Melo [15], reported that the cowpea (*Vigna unguiculata*) inoculation with bacterial strains*, Klebsiella trevisanii, Enterobacter agglomerans, Agrobacterium radiobacter,* and *Paracoccus denitrificans* associated with *Opuntia ficus-indica* having *nif*H gene significantly increases plant growth and more nodulation in secondary roots due to more surface area for easily colonization of *Rhizobium* spp. and *Bradyrhizobium* spp. de Lima [16] reported that the triple inoculation of cowpea seed with *Bradyrhizobium*, *Glomus,* and *Paenibacillus* enhances dry biomass and plant growth as well as nitrogen and phosphorus availability than control. Kanthaiah [17] stated that *Pseudomonas aeruginosa* VRKK1 isolated, biochemical and 16S rDNA gene sequencing from rhizosphere soil of cowpea, India. They tested growth promoting activity and extracted bioactive metabolite (octadecanoic acid 2-oxo methyl ester) as bio-controlling agent for 40.24% inhibition of disease severity of bacterial blight disease caused by *Xanthomonas campestris* in cowpea over control plants.

Presently, only single or dual microbial inoculants and non-indigenous/non-efficient strains are available in the market which are not effective for all vegetables. These inoculants isolated from different agroecological zone which doesn't colonize effectively in other soils regions. This is a major problem in bio-fertilizers/bio-inoculant availability in the markets for farmer's applications. For the solution to these types of problems, only indigenous microbial strains can easily colonize and sustain in local regions for plant growth and development. Therefore, the urgent need is to develop an efficient indigenous climate resilient microbial consortium for vegetable production. Here, we aimed to isolate and characterize the soil microbes from rhizosphere soils of vegetables for developing novel microbial consortia for enhancing lobia production.

## 2 Material and methods

### 2.1 Microbial enumeration & characterization

Soil samples were collected for microbial isolation from rhizosphere and non-rhizosphere soils of lobia growing field of Indian Institute of Vegetable Research Institute (IIVR) (GPS location: 25018'23.08"N; 82087'56.61"E), Varanasi, eastern Uttar Pradesh, India. Isolation of microbes was performed by serial dilution and plating method. 100 μL aliquot of each dilution was transferred and spread aseptically on Petri Plates containing respective media like Nutrient agar, King's B Base, Pikovskaya agar, Potato Dextrose agar and Tryptone Soya agar (Hi media Pvt. Ltd.). The plates were incubated for 2–5 days at 30 °C [8,9].

#### 2.1.1 Morphological and biochemical properties of microbes

**Fig. 1:**
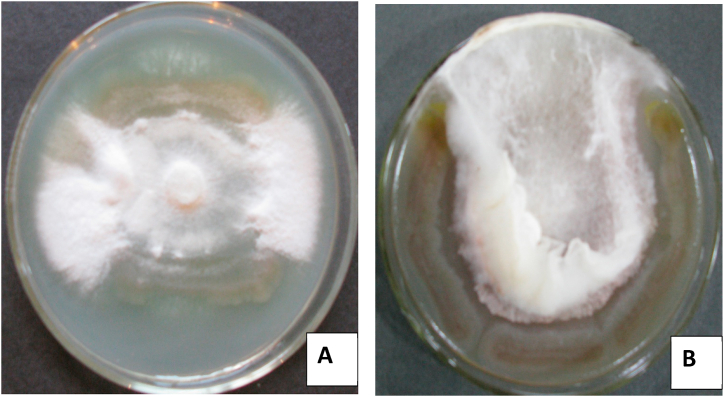
*Pseudomonas* sp. IESDJP-V1 showed inhibition zone against *Fusarium oxysporum* (A) and *Rhizoctonia solani* (B) on dual media plate of mixture of 50% nutrient agar and 50% Potato dextrose agar

Microbial strains were characterized based on morphological [18] and biochemical properties including cellulase and catalase production tests [18] (Table 1). The plant growth-promoting activities e.g., phosphate solubilization [19], indole-3-acetic acid (IAA) by using Salkowski reagent and a few drops of orthophosphoric acid [20], ammonia production by using Nessler's reagent [21], siderophore production by the use of CAS (Chrome Azurol S media) as described by Schwyn and Neilands [22] were estimated (Table 2). Bio-controlling activities was estimated on dual media plate by inoculation of *Fusarium oxysporum* and *Rhizoctonia solani* with bacterial strains [8] (Fig. 1A and B). Amylase test was done by using preparing 0.1% starch agar media, urease, citrate, and MRVP test was done [18].

**Table 1:** Biochemical characterization of isolated bacterial strain.

Strains	Biochemical characterization					
	Amylase	Catalase	Urease	Citrate test	Methyl red	Voges-Proskauer
*Pseudomonas* sp. IESDJP-V1	++	+++	–	+	–	–
*Pseudomonas* sp. IESDJP-V2	++	++	–	++	–	–
*Serratia marcescens* IESDJP-V3	++	+	+	+	–	+
*Bacillus cereus* IESDJP-V4	++	++	+	–	–	+
*Ochrobactrum* sp. IESDJP-V5	+	+	–	–	–	+
*Azospirillum brasilensis* MTCC-4037	+	+	+	+	+	–
*Paenibacillus polymyxa* BHUPSB17	+	++	–	+	–	–

*Note: In this table “+++”, “++”, “+” and “-”represent the production ability of microbes in high, moderate, low and absent, respectively. All experiment was conducted with 3 replications setup.

**Table 2:** Characterization of plant growth promoting biochemical activities of isolated strain.

Strains	Phosphate solubilization (μgml^−1^) at 3days	IAA production (μg ml^−1^) at 48 h		Siderophore production	Ammonia production	HCN production	Biocontrol activity	
		150 μgml^−1^ tryptophan	300 μgml^−1^ tryptophan				*Fusarium oxysporum*	*Rhizoctonia solani*
*Pseudomonas* sp IESDJP-V1	39.25 ± .66^e^	30.05 ± .86^f^	34.86 ± .17^e^	++	+++	++	+	++
*Pseudomonas* sp IESDJP-V2	33.02 ± .14^c^	18.27 ± .60^b^	32.06 ± .05^d^	+	++	+	+	++
*Serratia marcescens* IESDJP-V3	33.30 ± .16^c^	23.70 ± .35^d^	26.59 ± .07^c^	+	+	+	–	–
*Bacillus cereus* IESDJP-V4	37.48 ± .44^d^	20.08 ± .05^c^	25.23 ± .09^b^	+	+	+	+	+
*Ochrobactrum* sp IESDJP-V5	24.76 ± .12^b^	25.30 ± .87^e^	55.48 ± .08^g^	+	++	+	–	–
*Azospirillum brasilense* MTCC-4037	19.12 ± .12^a^	40.59 ± 1.18^g^	52.08 ± .13^f^	+	++	–	–	–
*Paenibacillus polymyxa* BHUPSB17	136.14 ± .10^f^	12.56 ± .18^a^	23.11 ± .03^a^	+	+	+	+	+

Note: The data Values are the mean ± SE, mean values in each column with the same superscript (s) do not differ significantly by Duncan multiple post hoc test (*P* = 0.05). The sign “+++”, “++”, “+” and “-” represent the production ability of microbes in high, moderate, low and absent, respectively. All experiment was conducted with 3 replications setup.

#### 2.1.2 Molecular identification of isolated microbial strains

Genomic DNA extraction was done by using methods described by Sambrook and Russel [23]. The universal Forward primer 27F and Reverse primer 939R were used for amplification of 16 S rDNA genes by PCR [8]. Amplification was performed by thermal cycler (BioRed). PCR product size was observed to be approximately 1000 bp (Thermo Scientific Pvt. Ltd.). PCR purification kit (Invitrogen, PureLink™ PCR purification kit, USA) was used for PCR product purification for sequencing of 16S rDNA gene. Gene sequencing of 16S rDNA was carried out BioKart India Pvt. Ltd. Bengaluru. The 16S rDNA sequences were matched with BLAST tool at NCBI for identification of bacteria and then it was submitted to NCBI GenBank for getting accession numbers (Table 3). The phylogenetic tree was constructed using mega version 4.1 by the neighbor-joining method [24].

**Table 3:** Molecular characterization of isolated bacterial strains.

Strains	Bacterial name	Accession Number isolated strains	Maximum identity (%)	Bacterial name of BLAST match
IESDJP-V1	*Pseudomonas* sp.	MH362754	99	*Pseudomonas* sp. strain P3 (MG75954)
IESDJP-V2	*Pseudomonas* sp.	MH362755	99	*Pseudomonas* sp. strain B15 (KY324872)
IESDJP-V3	*Serratia marcescens*	MH362756	99	*Serratia marcescens* strain YCGG3 (MF966252)
IESDJP-V4	*Bacillus cereus*	MH362757	99	*Bacillus cereus* strain LB7 (MF370350)
IESDJP-V5	*Ochrobactrum* sp.	MH362758	99	*Ochrobactrum* sp. Vr39 (LN851900)

### 2.2 Seed bacterization and pot trials on cowpea

**Fig. 2:**
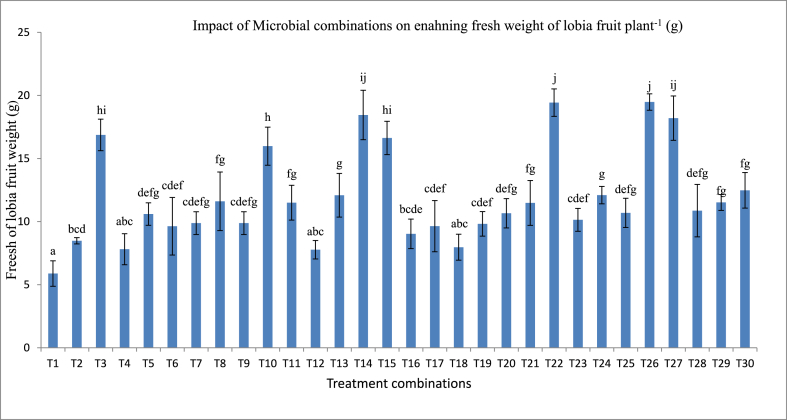
Effect of different microbial treatment on the fresh fruit weight of lobia (*Vigna unguiculata*). The data Values are the mean ± SE, mean values in each column with the same superscript (s) do not differ significantly by Duncan multiple post hoc test (*P*=0.05). Here, T1 is control and T2 to T30 are microbial treatments. All experiment was conducted with 3 replications setup.

**Fig. 3:**
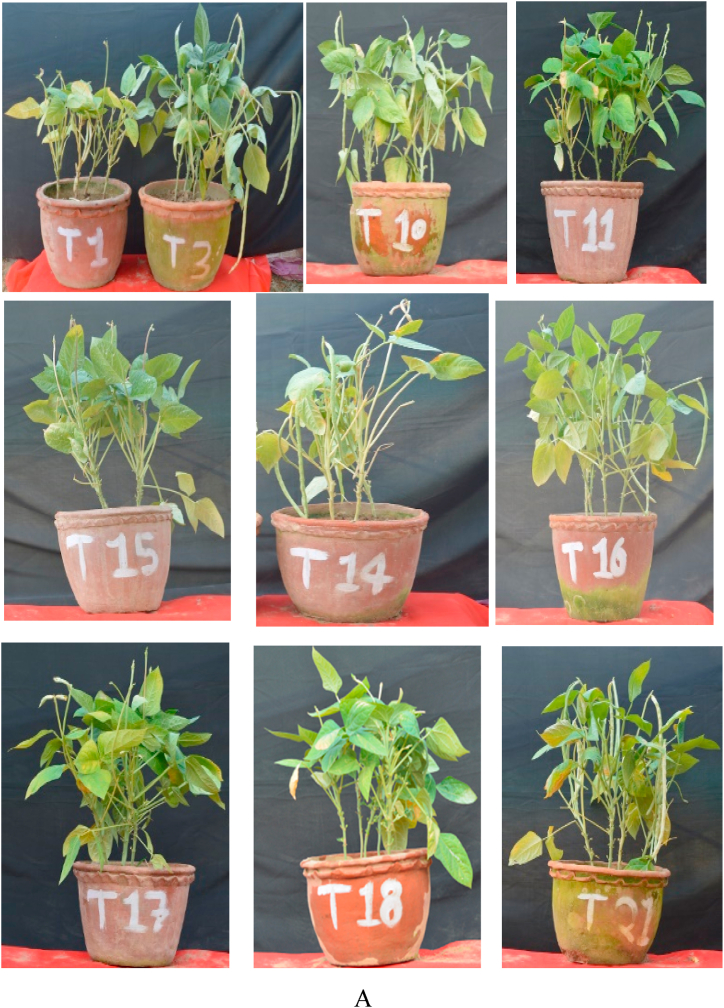

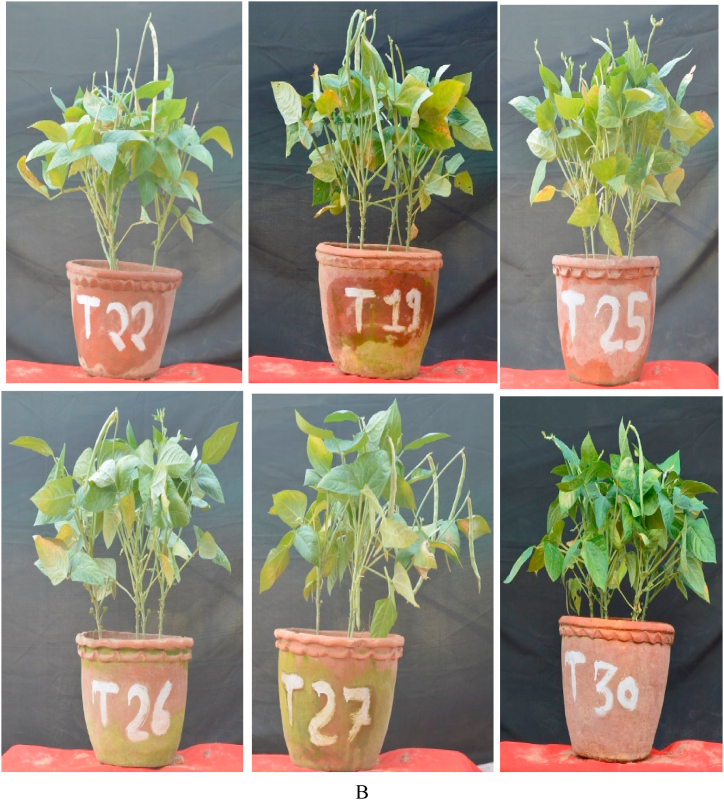
a. Pot experiment of lobia at IESD experimental plot showed very good comparison of different treatments of microbial consortium T3, T10, T11, T14, T15, T16, T17, T18 and T21 as compared to control T1.B Pot experiment of lobia at IESD experimental showed very good effect of microbial consortium in treatment combination T22, T19, T25, T26, T27 and T30 as compared to control T1

The pot experiment was conducted with 30 treatments combination in three replications including untreated control set on crops lobia (*Vigna unguiculata*) variety Kashi Kanchan in 2017 (Table 4). Thirty pots were prepared with 8 kg field soils amended with 20 g/kg farm yard manure (FYM). The surface sterilization was done by using 70% ethanol and 0.1% HgCl_2_ followed by washing in each step three times with sterilized distilled water [9]. Each bacterial culture was grown in nutrient broth liquid media at 150 rpm shaking BOD incubator at 30 °C for 72 h. Liquid broth culture of bacteria was used for seed inoculations. After seed sterilization, the seeds were mixed with 2% gum acacia in each treatment then added 5 mL bacterial broth culture (*Pseudomonas* sp. IESDJP-V1, *Pseudomonas* sp. IESDJP-V2, *Serratia marcescens* IESDJP-V3, *Bacillus cereus* IESDJP-V4, *Ochrobactrum* sp. IESDJP-V5, *Azospirillum brasilense* MTCC-4037, and *Paenibacillus polymyxa* BHUPSB17) volume having 10^8^ CFU/mL as per treatment wise. Each bacteria broth culture was mixed to make consortium as per treatment combinations in equal volume. After seed treatments, seeds were left for 1–2 h for further sowing in pot. After germination, the thinning was done to maintain two plants in each pot. The plant height, numbers of branches, and leaf data were taken after 30, 60 and 90 days. The experiment was conducted in three replications. The fresh weight of lobia fruit was taken from each treatment combination and calculated total production of fresh vegetables of lobia per plant in gram (Table 5, Fig. 2, Fig. 3A and B).

**Table 4:** Treatments combination of isolated microbial consortium for pot trials.

T1	Control
T2	*Pseudomonas* sp. IESDJP-V1
T3	*Pseudomonas* sp. IESDJP-V2
T4	*Serratia marcescens* IESDJP-V3
T5	*Bacillus cereus* IESDJP-V4
T6	*Ochrobactrum* sp. IESDJP-V5
T7	*Azospirillum brasilense*
T8	*Paenibacillus polymyxa*
T9	IESDJP-V1+ IESDJP-V2
T10	IESDJP-V2+ IESDJP-V3
T11	IESDJP-V1+ IESDJP-V4
T12	IESDJP-V1+ IESDJP-V5
T13	IESDJP-V1+ *Azospirillum brasilense*
T14	IESDJP-V2+ *Azospirillum brasilense*
T15	IESDJP-V1+ IESDJP-V2+ IESDJP-V3
T16	IESDJP-V1+ IESDJP-V2+ IESDJP-V4
T17	IESDJP-V1+ IESDJP-V2+ IESDJP-V5
T18	IESDJP-V1+ IESDJP-V2+ *Azospirillum brasilense*
T19	IESDJP-V1+ IESDJP-V2+ *Paenibacillus polymyxa*
T20	IESDJP-V1+ IESDJP-V3+ IESDJP-V4
T21	IESDJP-V1+ IESDJP-V3+ IESDJP-V5
T22	IESDJP-V1+ IESDJP-V3+ *Azospirillum brasilense*
T23	IESDJP-V1+ IESDJP-V3+ *Paenibacillus polymyxa*
T24	IESDJP-V1+ IESDJP-V4+ IESDJP-V5
T25	IESDJP-V1+ IESDJP-V4+ *Azospirillum brasilense*
T26	IESDJP-V1+ IESDJP-V4+ *Paenibacillus polymyxa*
T27	IESDJP-V1+ IESDJP-V5+ *Azospirillum brasilense*
T28	IESDJP-V1+ IESDJP-V5+ *Paenibacillus polymyxa*
T29	IESDJP-V1+ *Paenibacillus polymyxa* + *Azospirillum brasilense*
T30	IESDJP-V1+ IESDJP-V2+ IESDJP-V3+ IESDJP-V4+ IESDJP-V5

**Table 5:** Effect of microbial treatment on plant growth, branching and leaf in lobia (*Vigna unguiculata*).

Treatments	Height of Lobia (cm/plant)			Number of Branching plant^−1^			Number of Leaf's plant^−1^		
	30 days	60 days	90 days	30 days	60 days	90 days	30 days	60 days	90 days
T1	6.25 ± 0.5^abcd^	13.75 ± 3.1^a^	19.25 ± 2.6^ab^	3.50 ± 0.58^abcd^	7.00 ± 0.8^abc^	9.75 ± 2.1^abcd^	9.25 ± 1.0^a^	6.3 ± 7.5^a^	15.8 ± 1.5^a^
T2	4.75 ± 1.3^a^	14.75 ± 1.7^ab^	25.00 ± 3.4^cd^	4.00 ± 0.82^abcde^	7.25 ± 1.3^abc^	10.75 ± 1.0^abcd^	11.00 ± 2.7^ab^	15.3 ± 4.9^bcdef^	21.8 ± 3.8^bcd^
T3	6.50 ± 0.6^abcde^	19.00 ± 2.8^cdef^	25.50 ± 3.3^d^	3.00 ± 0.82^ab^	8.75 ± 2.2^bc^	13.75 ± 1.3^e^	8.50 ± 1.3^a^	17.8 ± 2.5^def^	24.3 ± 3.8^de^
T4	4.75 ± 1.7^a^	16.75 ± 4.1^bc^	20.75 ± 2.5^abcd^	4.00 ± 0.82^abcde^	8.50 ± 4.2^abc^	11.00 ± 3.7^bcde^	11.00 ± 1.8^ab^	13.0 ± 6.3^bcdef^	22.5 ± 9.3^cd^
T5	6.25 ± 1.7^abcd^	19.50 ± 3.3^cdef^	19.75 ± 3.5^abc^	4.25 ± 0.50^abcde^	8.75 ± 4.1^bc^	10.00 ± 0.0^abcd^	10.50 ± 0.6^a^	16.5 ± 3.0^cdef^	28.5 ± 9.0^e^
T6	5.75 ± 2.5^abc^	18.01 ± 1.6^cde^	19.00 ± 3.7^ab^	3.25 ± 0.50^abc^	8.25 ± 1.9^abc^	8.25 ± 1.9^abc^	9.75 ± 1.0^a^	15.3 ± 4.5^bcdef^	20.3 ± 2.9^abcd^
T7	6.00 ± 2.2^abcd^	18.25 ± 2.8^cde^	19.25 ± 4.3^ab^	4.00 ± 0.82^abcde^	8.50 ± 2.5^abc^	9.00 ± 1.2^abcd^	9.75 ± 2.2^a^	16.5 ± 1.7^cdef^	18.8 ± 1.5^abc^
T8	5.00 ± 2.2^ab^	15.25 ± 1.0^abc^	22.50 ± 6.2^bcd^	3.25 ± 0.50^abc^	6.75 ± 1.0^abc^	7.75 ± 2.6^a^	9.25 ± 0.5^a^	8.8 ± 4.6^ab^	18.0 ± 2.4^abcd^
T9	5.00 ± 1.6^ab^	17.25 ± 1.0^bcde^	21.00 ± 3.6^abcd^	2.75 ± 0.50^a^	6.75 ± 0.5^abc^	8.00 ± 1.6^ab^	9.00 ± 1.4^a^	11.8 ± 2.8^abcde^	20.3 ± 1.5^abc^
T10	5.50 ± 1.9^abc^	18.50 ± 3.1^cde^	21.25 ± 2.4^abcd^	2.75 ± 0.96^a^	6.25 ± 1.3^ab^	10.00 ± 0.0^abcd^	11.25 ± 1.9^ab^	13.8 ± 2.5^bcdef^	17.3 ± 1.5^abcd^
T11	4.75 ± 1.3^a^	21.50 ± 2.4^defg^	22.75 ± 2.2^bcd^	3.00 ± 0.82^ab^	7.00 ± 0.8^abc^	9.25 ± 4.3^abcd^	10.50 ± 1.3^a^	16.5 ± 1.7^cdef^	19.5 ± 3.0^abc^
T12	6.50 ± 1.0^abcde^	21.75 ± 2.2^defg^	22.00 ± 4.2^bcd^	4.00 ± 0.82^abcde^	7.50 ± 1.0^abc^	7.75 ± 2.9^a^	9.50 ± 1.0^a^	13.0 ± 2.9^bcdef^	18.0 ± 2.4^abc^
T13	5.00 ± 1.4^ab^	19.00 ± 2.2^cdef^	21.50 ± 3.1^abcd^	3.50 ± 0.58^abcd^	6.25 ± 1.3^ab^	8.50 ± 1.9^abc^	10.00 ± 2.2^a^	11.3 ± 7.5^abcd^	16.5 ± 3.0^ab^
T14	5.50 ± 1.7^abc^	17.50 ± 2.4^bcde^	23.75 ± 1.3^bcd^	6.00 ± 1.41^f^	8.75 ± 3.0^bc^	21.25 ± 1.7^f^	10.75 ± 2.2^a^	18.8 ± 2.9^e^	22.3 ± 1.5^cd^
T15	8.75 ± 1.7^de^	18.50 ± 1.9^cde^	21.50 ± 3.4^abcd^	5.00 ± 0.82^def^	6.25 ± 1.9^ab^	9.50 ± 1.0^abcd^	13.75 ± 4.2^b^	11.3 ± 2.5^abcd^	17.3 ± 1.5^abc^
T16	6.00 ± 0.8^abcd^	22.25 ± 4.8^g^	19.25 ± 4.7^ab^	3.75 ± 0.50^abcd^	7.50 ± 0.6^abc^	9.50 ± 1.0^abcd^	9.75 ± 0.5^a^	15.0 ± 0.0^bcdef^	16.5 ± 1.7^ab^
T17	9.25 ± 3.1^e^	21.50 ± 1.9^fg^	22.50 ± 0.6^bcd^	4.50 ± 0.58^bcde^	6.50 ± 1.0^ab^	9.50 ± 1.0^abcd^	10.75 ± 1.5^a^	12.0 ± 6.0^abcdef^	17.3 ± 2.9^abc^
T18	7.75 ± 2.1^bcde^	20.75 ± 8.5^efg^	22.75 ± 2.1^bcd^	3.50 ± 1.00^abcd^	6.50 ± 1.0^ab^	11.25 ± 2.5^cde^	10.25 ± 1.9^a^	13.8 ± 2.5^bcdef^	15.8 ± 1.5^a^
T19	6.00±.8^abcd^	20.00 ± 2.4^efg^	22.00 ± 2.2^bcd^	3.75 ± 0.50^abcd^	6.50 ± 1.0^ab^	9.00 ± 2.0^abcd^	9.00 ± 1.2^a^	15.0 ± 0.0^bcdef^	16.5 ± 3.0^ab^
T20	6.25 ± 1.3^abcd^	17.50 ± 0.6^bcde^	21.75 ± 1.9^abcd^	4.00 ± 1.15^abcde^	6.25 ± 0.1^ab^	10.00 ± 0.0^abcd^	9.75 ± 2.1^a^	9.0 ± 4.2^ab^	17.3 ± 2.9^abc^
T21	6.50 ± 1.0^abcde^	19.75 ± 2.6^cdef^	22.00 ± 3.9^bcd^	4.00 ± 0.82^abcde^	6.50 ± 0.6^ab^	10.00 ± 0.0^abcd^	10.50 ± 1.7^a^	12.3 ± 5.3^abcdef^	16.5 ± 1.7^ab^
T22	6.25 ± 2.6^abcd^	18.75 ± 3.9^cde^	23.25 ± 1.5^bcd^	4.00 ± 1.83^abcde^	7.00 ± 1.2^abc^	9.25 ± 1.0^abcd^	9.50 ± 2.9^a^	10.3 ± 5.9^abc^	18.8 ± 2.9^abc^
T23	6.50 ± 1.0^abcde^	20.25 ± 2.1^def^	20.50 ± 1.9^bcd^	4.25 ± 0.96^abcde^	6.00 ± 1.6^ab^	9.50 ± 1.0^abcd^	9.75 ± 0.5^a^	13.3 ± 3.5^bcdef^	18.8 ± 1.5^abc^
T24	6.00 ± 1.4^abcd^	18.50 ± 1.3^cde^	22.25 ± 2.9^bcd^	3.75 ± 1.26^abcd^	6.25 ± 0.5^ab^	10.75 ± 1.5^abcd^	9.00 ± 2.0^a^	16.5 ± 1.7^cdef^	18.0 ± 0.0^abc^
T25	7.25 ± 1.0^abcde^	20.50 ± 4.5^efg^	22.00 ± 1.6^bcd^	4.25 ± .96^abcde^	5.75 ± 0.5^a^	10.00 ± 0.0^abcd^	11.25 ± 2.5^ab^	17.3 ± 2.9^def^	22.5 ± 1.7^cd^
T26	8.00 ± 2.0^cde^	18.20 ± 1.5^cde^	25.00 ± 2.2^cd^	4.75 ± .96^cdef^	6.00 ± 0.2^ab^	13.50 ± 1.0^e^	10.00 ± 0.8^a^	13.8 ± 2.5^bcdef^	28.0 ± 0.0^e^
T27	8.25 ± 1.5^cde^	18.25 ± 2.8^cde^	25.75 ± 2.6^cd^	5.50 ± 1.00^def^	9.50 ± 1.0^c^	13.00 ± 0.0^e^	10.25 ± 2.1^a^	18.5 ± 3.3^ef^	27.3 ± 1.5^e^
T28	8.00 ± 2.2^cde^	18.50 ± 2.1^cde^	19.00 ± 4.2^ab^	3.25 ± 1.50^abc^	6.25 ± 0.5^ab^	9.50 ± 1.0^abcd^	8.50 ± 1.9^a^	13.8 ± 2.5^bcdef^	18.0 ± 0.0^abc^
T29	5.75 ± 1.0^abc^	17.75 ± 0.5^bcde^	18.00 ± 2.2^a^	4.25 ± 0.96^abcde^	8.25 ± 1.3^abc^	10.50 ± 3.3^abcd^	9.50 ± 1.0^a^	15.8 ± 1.5^cdef^	21.0 ± 2.4^abcd^
T30	5.50 ± 2.5^abc^	16.75 ± 2.1^bc^	23.75 ± 4.6^bcd^	4.25 ± 0.96^abcde^	6.75 ± 1.0^abc^	11.75 ± 2.1^de^	9.00 ± 2.0^a^	16.8 ± 1.5^cdef^	18.8 ± 1.5^abc^

Note: The data Values are the mean ± SE, mean values in each column with the same superscript (s) do not differ significantly by Duncan multiple post hoc test (*P* = 0.05). Here, T1 is control and T2 to T30 are microbial treatments. All experiment was conducted with 3 replications setup.

### 2.3 Assessment of fruits nutritional content

Total phenolic estimation was assayed as per the method described by Imeh and Khokhar [25]. The phenolics concentrations in pod samples were expressed as milligrams of catechol equivalent per gram fresh weight of the samples. The estimation of total flavonoids was done according to Chang et al. [26] and it was expressed in milligrams (mg) of quercetin equivalent per gram (g) of sample. The total protein estimation was done as per the method described previously [27]. Total protein concentrations were expressed in milligrams (mg) of protein equivalent per gram (g) of the sample (Table 6). Total sugar (reducing and non-reducing) was estimated by DNS (dinitrosalicylic acid) method [28]. The non-reducing sugar was estimated as per method of Malhotra and Sarkar [29]. Total sugar was calculated in grams per 100 g fresh weight (Table 6).

**Table 6:** Effect of microbial treatments on nutritional content of lobia fruits under pot trials.

Treatments	Nutritional content of Lobia fruit					
	Protein (mg/gram fresh weight)	Phenol (mg/gram fresh weight)	Reducing Sugar (gm/100gm fresh weight)	Non-Reducing Sugar (gm/100 gm)	Total Sugar (gram/100 g)	Flavonoids (mg/gram dry weight)
T1	46.69±0.61^a^	4.01±0.03^a^	0.86±0.01^a^	0.59±0.02^a^	1.43±0.03^a^	0.40±0.02^a^
T2	51.25±1.72^b^	4.30±0.20^ab^	0.89±0.03^abc^	0.66±0.02^b^	1.53±0.02^b^	0.45±0.04^b^
T3	52.68±1.07^def^	4.56±0.35^b^	0.91±0.02^abcde^	0.67±0.03 ^bcdefg^	1.57±0.02^bc^	0.50±0.02^c^
T4	51.53±0.49^ab^	5.10±0.09^cdefg^	0.91±0.04^abcde^	0.66±0.02^bc^	1.58±0.04^bcd^	0.45±0.02^b^
T5	53.23±0.33^efghij^	5.20±0.15^cdefg^	0.87±0.04^ab^	0.70±0.02^cdefgh^	1.58±0.01^bc^	0.50±0.02^c^
T6	52.60±0.54^def^	4.91±0.07^c^	0.92±0.03^abcde^	0.69±0.02^bcde^	1.61±0.02^bcde^	0.51±0.02^c^
T7	53.39±0.23^efghij^	5.00±0.11^cde^	0.87±0.02^ab^	0.67±0.02^bcde^	1.57±0.01^bc^	0.50±0.02^c^
T8	52.98±0.03^defghi^	4.95±0.16^cd^	0.89±0.03^abcd^	0.71±0.02^defgh^	1.61±0.01^bcde^	0.49±0.03^ab^
T9	53.90±0.10^ij^	5.08±0.15^cdef^	0.92±0.02^abcde^	0.72±0.03^fgh^	1.64±0.02^abcdef^	0.50±0.01^c^
T10	52.79±0.19^defg^	5.09±0.20^cdef^	1.00±0.04^cdefghi^	0.71±0.02^defgh^	1.70±0.02^degh^	0.49±0.02^ab^
T11	52.46±0.58^cde^	4.99±0.11^cde^	0.99±0.03^cdefgh^	0.72±0.03^fgh^	1.73±0.06^efghi^	0.52±0.03^c^
T12	53.41±0.51^efghij^	5.36±0.57^fg^	0.95±0.02^abcdefg^	0.69±0.02^bcdefgh^	1.63±0.01^bcde^	0.53±0.03^c^
T13	53.53±0.55^fghij^	5.24±0.06^cdefg^	0.94±0.03^abcdef^	0.69±0.04^bcdefgh^	1.63±0.03^bcdef^	0.52±0.01^c^
T14	54.10±0.45^j^	5.24±0.12^cdefg^	0.98±0.03^bcdefgh^	0.71±0.02^defgh^	1.87±0.06^jkl^	0.53±0.03^c^
T15	52.95±0.24^defghi^	5.14±0.06^cdefg^	1.01±0.05^efghij^	0.71±0.02^efgh^	1.71±0.01^efgh^	0.53±0.03^c^
T16	52.98±0.63^defghi^	5.33±0.23^efg^	1.01±0.02^efghij^	0.71±0.02^efgh^	1.70±0.02^efgh^	0.51±0.02^c^
T17	53.41±0.52^efghij^	5.40±0.35^fg^	1.02±0.05^efghij^	0.72±0.03^fgh^	1.71±0.03^efgh^	0.51±0.01^c^
T18	53.84±0.21^hij^	5.08±0.13^cdef^	1.01±0.04^efghij^	0.70±0.02^cdefgh^	1.71±0.01^efgh^	0.52±0.03^c^
T19	53.73±0.07^ghij^	5.18±0.08	1.06±0.12^ghijkl^	0.72±0.03^fgh^	1.80±0.12^ghijk^	0.49±0.01^ab^
T20	53.88±0.14^hij^	5.14±0.08^cdefg^	1.17±0.07^lmn^	0.73±0.03^h^	1.68±0.04^cdeg^	0.50±0.04^c^
T21	53.77±0.12^ghij^	5.19±0.06^cdefg^	1.17±0.11^mn^	0.71±0.03^efgh^	1.88±0.14^kl^	0.50±0.02^c^
T22	53.95±0.06^ij^	5.21±0.14^cdefg^	1.20±0.18^m^	0.73±0.03^gh^	1.86±0.02^jkl^	0.49±0.01^ab^
T23	53.76±0.55^ghij^	5.41±0.13^fg^	1.05±0.10^fghijk^	0.70±0.02^cdefgh^	1.75±0.10^fghij^	0.50±0.02^c^
T24	52.75±0.66^defg^	5.45±0.09^g^	1.14±0.02^klmn^	0.72±0.02^fgh^	1.68±0.03^cdeg^	0.50±0.02^c^
T25	52.46±0.18^cde^	5.35±0.05^efg^	1.12±0.02^jklmn^	0.72±0.01^fgh^	1.83±0.02^ijkl^	0.49±0.03^ab^
T26	54.84±0.25^j^	5.45±0.09^g^	1.21±0.08^m^	0.74±0.02^h^	1.96±0.11^l^	0.54±0.06^c^
T27	53.99±0.12^ij^	5.37±0.10^fg^	1.00±0.03^cdefghi^	0.71±0.02^efgh^	1.93±0.21^l^	0.53±0.02^c^
T28	53.25±0.35^efghij^	5.09±0.14^cdef^	0.98±0.02^bcdefgh^	0.68±0.01^bcdef^	1.69±0.01^cdeg^	0.50±0.03^c^
T29	52.70±0.30^def^	5.20±0.01^cdefg^	1.00±0.04^defghi^	0.72±0.02^fgh^	1.69±0.04^cdeg^	0.50±0.03^c^
T30	52.19±0.21^bc^	5.22±0.09^cdefg^	1.11±0.02^ijklmn^	0.70±0.01^cdefgh^	1.82±0.03^hijkl^	0.51±0.01^c^

Note: The data Values are the mean ± SE, mean values in each column with the same superscript (s) do not differ significantly by Duncan multiple post hoc test (*P*=0.05). Here, T1 is control and T2 to T30 are microbial treatments. All experiment was conducted with 3 replications setup.

### 2.4 Physico-chemical properties of pot soil sample after experiment

Soil samples were collected from the experimental pot for estimation of soil pH, EC, and organic carbon. pH was measured in a water suspension (1:2.5) (10 g soil with 25 mL of sterilized distilled water) [30]. This suspension was also used for estimation of the electrical conductivity (EC) by EC meter and expressed as μS cm^−1^ [31]. Soil organic carbon and organic matter were estimated by modified methods of Walkley and Black [32] (Table 7).

**Table 7:** Effect of microbial treatment on soil properties of pot experiment

Chemical properties of pot experiment soil			
Treatments	Soil pH	Soil EC (μS cm^-1^)	Organic Carbon (%)
T1	8.6±0.00^a^	90.5±0.50^a^	0.3±.27^ab^
T2	8.3±0.30^a^	191.5±2.50^op^	0.3±.31^a^
T3	8.4±0.25^a^	133.0±4.00^g^	1.0±.03^abcdef^
T4	8.3±0.30^a^	120.0±1.00^d^	1.1±.03^cdefg^
T5	8.3±0.30^a^	90.5±0.50 ^a^	1.1±.03^cdefg^
T6	8.2±0.25^a^	110.5±1.50^c^	0.6±.03^abcd^
T7	8.6±0.00^a^	197.0±2.00^p^	0.6±.17^abcd^
T8	8.3±0.30^a^	111.0±3.00^c^	0.3±.14^ab^
T9	8.3±0.30^a^	127.0±14.00^ef^	0.4±.14^abc^
T10	8.6±0.05^a^	191.0±2.00^o^	1.1±.35^defg^
T11	8.3±0.35^a^	102.5±4.50^b^	0.4±.34^abc^
T12	8.2±0.20^a^	141.5±0.50^hi^	1.3±.07^efgh^
T13	8.6±0.00^a^	166.0±2.00^m^	1.0±.51^bcdef^
T14	8.6±0.00^a^	159.5±0.50^l^	0.6±.33^abcde^
T15	8.6±0.00^a^	177.5±2.50^n^	1.7±.00^jk^
T16	8.3±0.25^a^	152.0±4.00^k^	1.6±.36^jk^
T17	8.3±0.30^a^	136.5±1.50^gh^	1.8±.56^ghijk^
T18	8.4±0.28^a^	144.5±1.50^i^	1.2±.21^defg^
T19	8.4±0.15^a^	150.5±0.50^jk^	1.1±.12^cdefg^
T20	8.3±0.30^a^	102.0±1.00 ^b^	1.7±.00^ghij^
T21	8.3±0.30^a^	97.0±1.00 ^b^	1.1±.07^defg^
T22	8.5±0.17^a^	131.0±2.00^fg^	1.1±.12^defg^
T23	8.4±0.35^a^	100.5±2.50 ^b^	1.4±.12^efgh^
T24	8.4±0.17^a^	123.5±1.50^de^	1.8±.81^jk^
T25	8.5±0.10^a^	140.5±0.50^hi^	1.0±.64^bcdef^
T26	8.4±0.26^a^	170.0±5.00^m^	1.6±.59^ghij^
T27	8.4±0.16^a^	125.5±2.50^def^	1.9±.75^k^
T28	8.4±0.26^a^	146.0±1.00^ij^	1.5±.86^fghij^
T29	8.6±0.18^a^	142.0±0.00^hi^	1.5±.59^ghij^
T30	8.5±0.19^a^	133.5±2.50^g^	1.7±.00^ghij^

Note: The data Values are the mean ± SE, mean values in each column with the same superscript (s) do not differ significantly by Duncan multiple post hoc test (*P*=0.05). Here, T1 is control and T2 to T30 are microbial treatments. All experiment was conducted with 3 replications setup.

### 2.5 Statistical analysis

Thirty treatments with three replications were used for experimental setup. Data was represented mean ± SE (standard error). Data was analyzed by analysis of variance (ANOVA) using Duncan post hoc multiple comparison tests. The SPSS software (version 20.0) was used for data analysis at significant value *P* ≤ 0.05. Principal Component analysis (PCA) was done by the statistica software.

## 3 Results

### 3.1 Bacterial isolation and their biochemical and PGPR properties

A total of 50 strains were isolated from rhizosphere soils of lobia. Out of 50, only 5 bacterial strains were characterized based on morphological and plant growth-promoting activities. The morphological and biochemical characterization had done and found that most of the microbes can produce amylase, catalase, and citrate and few microbes produce MR, VP, and citrate. Five selected bacterial strains recorded positive for amylase, catalase, and citrate test, while *B. cereus* IESDJP-V4 and *Ochrobactrum* sp. IESDJP-V5 showed negative citrate test. Strain IESDJP-V1, IESDJP-V2, IESDJP-V5 and BHUPSB17 showed negative for urease production than others. *A. brasilense* gave positive for methyl red while others showed negatives. Strains IESDJP-V3, IESDJP-V4, and IESDJP-V5 showed positive tests for Voges-Proskauer except for others (Table 1). Phosphate solubilization was recorded significant more 136.14 μg mL^−1^ in bacteria strain *P. polymyxa* BHUPSB17 followed by BHUJPV-V1, IESDJP-V4, IESDJP-V3 & IESDJP-V2 than IESD-JP-V5 and MTCC-4037 at 3 days. IAA production by *A. brasilense* MTCC-4037 (40.59 & 52.08 μg mL^−1^), *Ochrobactrum* sp. IESDJP-V5 (25.30 & 55.48 μg mL^−1^) and *Pseudomonas* sp. IESDJP-V1 (25.30 and 55.48 μg mL^−1^) followed by *Pseudomonas* sp. IESDJP-V1 (18.27 & 32.06 μg mL^−1^) and *S. marcescens* IESDJP-V3 (23.70 & 26.59 μg mL^−1^) as compared to *P. polymyxa* BHUPSB17 (12.56 & 23.11 μg mL^−1^) at 48 h at 150–300 μg mL^−1^ tryptophan concentration as IAA precursor in broth media, respectively. All strains showed positive for siderophore, ammonia, and HCN production while the potential strain *Pseudomonas* sp. IESDJP-V1 was recorded more than others (Table 2). *Pseudomonas* sp. IESDJP-V1, IESD-2, *Bacillus cereus* IESD-V4 and *P. polymyxa* BHUPSB17 showed inhibition zone against soil borne phytophatogenic fungi e.g., *Fusarium oxysporum* and *Rhizoctonia solani* as compared to others strains (Fig. 1A and B). These bacterial strains can possible to use as bio-controlling agent for fungal pathogen in soils and plants. The bacterial strains e.g., IESDJP-V1, IESDJP-V2, IESDJP-V3, IESDJP-V4, and IESDJP-V5 were selected for molecular characterization and used as bio-inoculants for further pot experiment. Molecular characterization of isolated microbes through 16s rDNA sequencing showed that all five microbes belong to the family of *Pseudomonas* sp. (IESDJP-V1 and V2), *S. marcescens* (IESDJP-V3), *B. cereus* (IESDJP-V4), *Ochrobactrum* sp. (IESDJP-V5). These strains were submitted in NCBI-GenBank and got accession number of Pse*udomonas* sp. IESDJP-V1 (MH362754) and *Pseudomonas* sp. IESDJP-V2 (MH362755), *S. marcescens* IESDJP-V3 (MH362756), *B. cereus* IESDJP-V4 (MH362757), *Ochrobactrum* sp. IESDJP-V5 (MH362758) (Table 3).

### 3.2 The effect of microbial consortium on plant growth attributes and yields on lobia

Plant height, branching, and number of leaves data were recorded at 30, 60, and 90 days intervals (Table 5, Fig. 2, Fig. 3A and B). The data showed the plant attributes were enhanced in many treatment combinations as compared to control plants. The plant height at 90 days was observed significant increase in the different treatment combinations but maximum height was observed in T27 (*Pseudomonas* sp. IESDJP-V1+ *Ochrobactrum* sp. IESDJP-V5+ *A. brasilense*) (0.33 fold), T3 (*Pseudomonas* sp. IESDJP-V2) (0.32 fold) and T26 (*Pseudomonas* sp. IESDJP-V1+ *B. cereus* IESDJP-V4+ *P. polymyxa*) (0.29 fold) than control plant growth. The number of branching at 90 days were highest in the treatment combination of T14 (*Pseudomonas* sp. IESDJP-V2 + *A. brasilense*) (1.17 fold), T3 (0.41 fold), T26 (0.38 fold) and T27 (0.33 fold) than control. The number of leaves after was recorded significant increase in T5 (*Bacillus cereus* IESDJP-V4) (0.80 fold), T26 (0.77 fold) and T27 (0.72 fold) followed by T3 (0.53 fold) than control at 90 days plant growth (Table 5, Fig. 2, Fig. 3A and B).

In the case of the fruit yield, all treatment combinations showed significant increase in fruit yields than control except for treatment combinations of T4, T12, and T18. The highest yield was observed in the treatment combination of T26 (*Pseudomonas* sp. IESDJP-V1+ *B. cereus* IESDJP-V4 + *P. polymyxa*), T22 (*Pseudomonas* sp. IESDJP-V1+ *S. marcescens* IESDJP-V3+ *A. brasilense*), T14 (*Pseudomonas* sp. IESDJP-V2 + *A. brasilense*), T3 (*Pseudomonas* sp. IESDJP-V2) and T27 (*Pseudomonas* sp. IESDJP-V1+ *Ochrobactrum* sp. IESDJP-V5+ *A. brasilense*) followed by T15 (*Pseudomonas* sp. IESDJP-V1+ *Pseudomonas* sp. IESDJP-V2+ *S. marcescens* IESDJP-V3) and T10 (*Pseudomonas* sp. IESDJP-V2+ *S. marcescens* IESDJP-V3) as compared to control and other treatment combinations (Figs. 2 and 3A and B).

Overall, the effect of seed inoculation on plant height, number of branching, leaf, and yield were observed in various treatment combinations and some treatment combinations showed good results in plant growth. Overall, the treatment combination of T3, T14, T26 and T27 (showed more significant results in plant height, branching and productivity as compared to control (Table 5).

### 3.3 The effect of microbial consortium on nutritional content in fruits

The crude protein content in fruit of lobia was estimated more in treatment T26 (0.17 fold) and T14 (0.16 fold), T27 (0.15 fold) followed by T22 (*Pseudomonas* sp. IESDJP-V1+ *S. marcescens* IESDJP-V3+ *A. brasilense*) (0.15 fold) as compared to control and other treatments (Table 6). Similarly, all treatment combinations showed significant increase in phenol as compared to control, and maximum phenol content was observed in the treatment of T24 (*Pseudomonas* sp. IESDJP-V1+ *B cereus* IESDJP-V4 + *Ochrobactrum* sp. IESDJP-V5) (0.35 fold) and in T26 (0.35 fold) followed by T27 (0.33 fold) than control and other treatments. The reducing sugar in fresh fruit of lobia was estimated highly significant content in reducing sugar in T26 (0.40 fold) and T22 (*Pseudomonas* sp. IESDJP-V1+ *S. marcescens* IESDJP-V3+ *A. brasilense*) (0.39 fold) as compared to control and others treated plants. The non-reducing sugar concentration in lobia fruit was found significant enhancement in all treatment combinations of microbial consortium as compared to control. Treatment combination T26 (*Pseudomonas* sp. IESDJP-V1+ *B. cereus* IESDJP-V4 + *P. polymyxa*) showed maximum non-reducing (0.25 fold) where T22 (*Pseudomonas* sp. IESDJP-V1+ *S. marcescens* IESDJP-V3+ *A. brasilense*) and T20 (*Pseudomonas* sp. IESDJP-V1+ *S. marcescens* IESDJP-V3+ *B. cereus* IESDJP-V4) contain same amount of non-reducing sugar (0.23 fold) as compared to untreated control and others treatments. Total sugar in lobia also showed significant in all treatment combinations than control. The highest significant enhancement of total sugar was recorded in treatment combination of T26 (0.37 fold), T27 (0.34 fold) as compared to controls and others. The flavonoids concentration estimated quantitatively and found that the treatment combinations e.g., T26 (0.35 fold), T15 (*Pseudomonas* sp. IESDJP-V1+ *Pseudomonas* sp. IESDJP-V2+ *S. marcescens* IESDJP-V3) (0.32 fold) and T14 (*Pseudomonas* sp. IESDJP-V2 + *A. brasilense*) (0.32 fold) showed maximum increase as compared to controls and others. The nutritional content of all treatment combination was generally found to increase as compared to control. The effective treatment combination T14, T22, T26 and T27 were found significant enhancement of protein, phenol, sugar, and flavonoids in fruits of lobia as compared to control and others (Table 6).

### 3.4 The effect of microbial consortium on soil properties after experiment

The soil pH of each treatment combination showed no significant increase or decrease during pot experiment soils. The EC (electrical conductivity) of the soils were found significant increase in all treatment of soil as compared to control. The highest significant increase of EC was observed in treatment combination of T7 (*A. brasilense*) (1.17 fold) than control and other treated soil samples. The organic carbon of soils is the most important factor, which depends on organic matter present in soils. This experiment was conducted in pot and this 20 g/kg farm yard manure was added in soils. The highest significant enhancement of organic carbon was recorded in treatment combinations T27 (*Pseudomonas* sp. IESDJP-V1+ *Ochrobactrum* sp. IESDJP-V5+ *A. brasilense*) (5.33 fold) than control and other treatments. During pot experiments, leaf litter, root exudes, and microbial population growth may be played a significant role in enhancing the organic carbon percentage in different treatments of soils (T9). The soil properties e.g., pH, EC, and organic carbon were found generally increase in more treatments than control and others (Table 7).

**Fig. 4:**
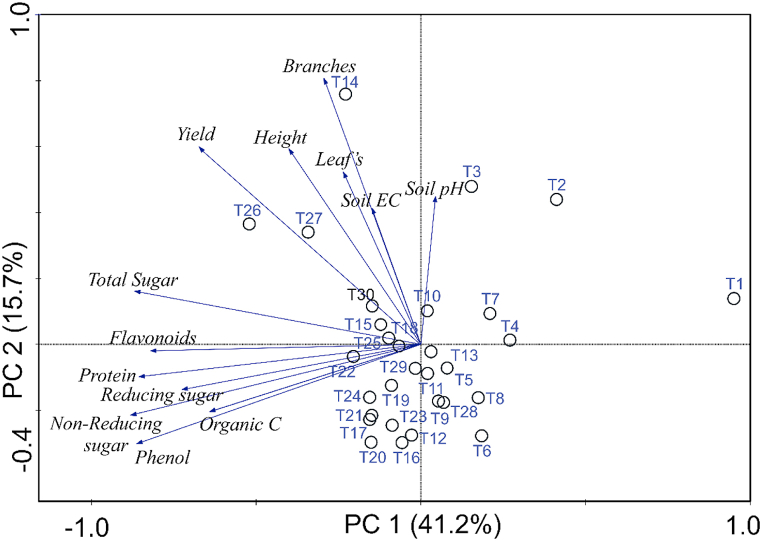
Principal component analysis (PCA) among all treatments with respect of plant growth attributes, yields, nutritional content and soil properties

Principal component analysis (PCA) was done and correlation was observed among the plant growth attributes, nutrient content and soil properties. T26 (*Pseudomonas* sp. IESDJP-V1+ *B. cereus* IESDJP-V4 + *P. polymyxa*) and T27 (*Pseudomonas* sp. IESDJP-V1+ *Ochrobactrum* sp. IESDJP-V5+ *A. brasilense*) were found more closely positive correlation with yield data of lobia. After that T14 (*Pseudomonas* sp. IESDJP-V2 + *A. brasilense*), T3 (*Pseudomonas* sp. IESDJP-V2), T10 (*Pseudomonas* sp. IESDJP-V2+ *S. marcescens* IESDJP-V3) followed by T15 (*Pseudomonas* sp. IESDJP-V1+ *Pseudomonas* sp. IESDJP-V2+ *S. marcescens* IESDJP-V3) and T30 (IESDJP-V1+ IESDJP-V2+ IESDJP-V3+ IESDJP-V4+ IESDJP-V5) were found positive correlation with yield data, plant height, branches, total sugar as compared to control T1 and others treatments. Others treatments were recorded negative correlation with yields, plant height, branching and nutritional contents (Fig. 4).

## 4 Discussion

Plant growth-promoting rhizobacteria is free-living microbes residing in rhizosphere and non-rhizosphere which have the ability to secret plant growth-promoting compounds for enhancing plant growth and development. In this study, all bacterial isolates showed positive for phosphate solubilization, IAA production, ammonia, siderophore, and HCN production except *A. brasilense* which showed negative HCN. Strains e.g, IESDJP-V1, IESD-2, IESD-V4 and BHUPSB17 showed positive for inhibition zone against soil borne phytophatogenic fungi e.g., *Fusarium oxysporum* and *Rhizoctonia solani* (Fig. 1A and B). These bacterial strains can possible to use as bio-controlling agent for fungal pathogen in cowpea (Table 2). Parasuraman et al. [33] isolated *Pseudomonas aeruginosa* strain JB and JC from rhizosphere of brinjal (*Solanum melongena* L.) and chilli (*Capsicum annuum* L.) which showed significant enhancement of seed germination and growth of *S. lycopersicum* (L.) due plant growth promoting compounds as phytohormones and salicylic acid under pot trials. Also these strains recorded significant inhibition of soil borne phytopathogenic fungi e.g., *Fusarium oxysporum* and *Alternaria solani*. The 16s rRNA gene sequencing of isolated microbes showed that *Pseudomonas* was the dominant species among 5 isolates. As per result of thirty treatment combinations of 7 bacterial strains, the treatment combination of T3 (*Pseudomonas* sp. IESDJP-V2), T14 (*Pseudomonas* sp. IESDJP-V2 + *A. brasilense*), T26 (*Pseudomonas* sp. IESDJP-V1+ *B. cereus* IESDJP-V4+*P. polymyxa*) and T27 (*Pseudomonas* sp. IESDJP-V1+ *Ochrobactrum* sp. IESDJP-V5+ *A. brasilense*) showed more significant results in plant height, branching, number of leaves, productivity, nutritional quantity and soil fertility as compared to control and others combinations (Table 5, Table 6 &7; Fig. 1). Similar findings reported by Rocha et al. [34], who observed that seed coating with *P. libanensis* and *R. irregularis* found significant enhancement in plant dry weight (76%) and grain yield (56%) than control plants of cowpea. Also, Souza-Alons et al. [35], reported that alginate-coated cells of *P. libanensis* inoculated with *V. unguiculata* observed more viability of bacterial cells in soils and provide sustainability against drought stress and enhance plant productivity and plant growth. Dizotrophic bacterial seed inoculation and AM inoculum with *Vigna unguiculata* (L.) enhanced plant tolerance, productivity and grain nutrient content (Nitrogen and Phosphorus), plant growth and development [14,36]. PGPR may promote plant growth and development through phytohormones production, nutrient solubilization, and improving defense systems of host plants by lowering ethylene levels in plants [2,5,6,10,[36], [37], [38]]. Wang et al. [51] observed that impact of PGPR strain Bacillus amyloliquefaciens SQR9 with and without inoculation with foliar application of amino acid liquid fertilizer enhanced cowpea yields and leaf microbiome as compared to control (chemical fertilizer), this experiment supports our research finding. The foliar sprayed of PGPR can be showed positive impact because colonization of beneficial microbe [6,9,39,40]. Hence; more research should be required to give the clear justification about impact and significant of PGPR and its plant growth promoting properties (IAA, siderophore, ammonia, phosphate solubilization and bio-control properties).

The indole-3-acetic acid (IAA) like other plants hormones biosynthesis may occur various metabolic pathways in plant tissues which promotes plant biomass [5,7]. PGPR can synthesize plant growth-promoting compounds which are very important for increasing soil fertility and health [12,[41], [42]]. PGPR affects auxin concentration in plant which stimulate nodulation initiation [43,44] and promote genes expression in the *Rhizobium*-legume interaction for symbiotic associations. The symbiotic association between leguminous plant with rhizobia help for biological nitrogen fixation that enhance plant growth attributes and grain yield [45,46].

Among all the soil physio-chemical properties, EC and OC were significantly changed in all treatment combinations. This result from the pot soil indicates that the microbes and their consortium help to induce the soil fertility by the change of EC and OC as these were the important soil health indicators for crop productivity [6,47]. Electrical conductivity or EC is one of the important soil chemical properties that are directly linked with the amount of soil ions. The results of our present research study explore the effects of PGPR both on plant health, yield and soil fertility and health. PGPR consortium promotes plant health and crop productivity due to direct and indirect mechanisms like plant hormones production, nutrient solubilization of phosphate, and potassium and production of siderophore, ammonia, suppression of phytopathogens [2,9]. PGPR mechanisms are helpful to increase soil fertility and health which depends on soil EC, OC, OM etc. [48].

Our isolated bacterial strains have multiple plant growth promoting properties such as plant growth hormone IAA, solubilization of the plant growth minerals (phosphate), ammonia production which directly supply the nitrogen to the host plant for the growth. Beside the growth promoting ability, isolated strains have ability to show HCN and pathogen inhibition due to may be production of volatile compound and chitinase production or may be production of antibiotic/antifungal secondary metabolites which provide protection to plant with different soil borne phytopathogen. As per the literate, similar type of bacterial strains produces or release many different types of metabolites that promote the plant growth and also protect them from various disease. Rangel-Montoya et al. [49], showed the biocontrol mechanism against the *Macrophomina phaseolina* in Cowpea plant by using *B. amyloliquefaciens,* with similar type of biocontrol property which present in our microbes. Some study supports our result to improve the nutrient value in the crops as compared to control [50,51]. In some combination of microbial treatment showed improvement in plant growth parameter as it clearly showed that microbes and consortia able to secret plant growth promoting factors. Where some other combinations don't show very fruitful result, as we can conclude that may the microbial consortia are not able to colonised properly with host plant, or may some environmental factor can hamper the growth of that microbes and its consortia. Different environment and soil condition, nutrient availability can hinder the proper growth of the microbes, from this we can say that some of our microbes and its consortia don't showed significant result during our experiment. Other reason of non-effective treatment combination due to possibility of nutrient limiting factor and compatibility with soil microbes it may be antagonistic to supress the growth of introduce cultures. Therefore, PGPR having multiple plant growth promoting properties can be used in form of consortium which gives cumulative impact on plant growth promotion and productivity as well as soil fertility and health.

## 5 Conclusion

The effective treatment T3 (*Pseudomonas* sp. IESDJP-V2), T14 (*Pseudomonas* sp. IESDJP-V2 + *A. brasilense*), T26 (*Pseudomonas* sp. IESDJP-V1+ *B. cereus* IESDJP-V4+*P. polymyxa*) and T27 (*Pseudomonas* sp. IESDJP-V1+ *Ochrobactrum* sp. IESDJP-V5+ *A. brasilense*) showed more significant results in plant height, branching, number of leaves and yield as compared to control and others. Finally, treatment combination T27 (*Pseudomonas* sp. IESDJP-V1+ *Ochrobactrum* sp. IESDJP-V5+ *A. brasilense*), T26 (*Pseudomonas* sp. IESDJP-V1+ *B. cereus* IESDJP-V4 + *P. polymyxa*) were found the effective microbial consortium for sustainable lobia production. Both combinations also showed more positive correlation for yield in prinicipal component analysis. Other combination of treatments was not found significant result due to possibility of nutrient limitation and negative compatibility of our consortium with natural soil microbores. In future, these consortia may be tested in different agro-ecological zone and climatic condition to evaluate real impact analysis at farmer fields. These consortia could be increased yields up to 25–30% in different vegetable crops in eastern Uttar Pradesh under field trials. This consortium can enhance the nutrition quality of lobia, soil fertility, and health. This consortium will be eco-friendly, cost-effective, and socially acceptable. It can help to reduce mineral fertilizer application. The limitation of this study is to need for conducting field trial with more replications to get more appropriate result for interpretations. Others limitation, the study of microbial colonization in rhizosphere and molecular mechanism of plant-microbe interaction through use of next generation sequencing (NGS) for metagenomic and rhizosphere microbiome analysis needs.

## Author contribution statement

Prakash Verma and Durgesh Kumar Jaiswal: Conceived and designed the experiments.

Durgesh Kumar Jaiswal and Anand Kumar Gaurav: Performed the experiments.

Ram Krishna, Arpan Mukherjee, Jay Prakash Verma and Arthur Prudêncio de Araujo Pereira: Analyzed and interpreted the data.

Durgesh Kumar Jaiswal, Arthur Prudêncio de Araujo Pereiraand Anand Kumar Gaurav: Contributed reagents, materials, analysis tools or data.

Gaurav: Wrote the paper.

Durgesh Kumar Jaiswal, Jay Prakash Verma, Arpan Mukherjee, Ram Krishna, Arthur Prudêncio de Araujo Pereira, Anand Kumar.

## Funding statement

Professor Jay Prakash Verma was supported by 10.13039/501100001843Science and Engineering Research Board (SERB), New Delhi [SIR/2022/000626], 10.13039/501100021098Design and Innovation Centre, BHU and Indian Institute of Technology (IIT-BHU) Varanasi [DIC-BHU/Project S-23 Approval/2016-17/693].

## Data availability statement

Data associated with this study has been deposited at NCBI-GenBank and got accession number of Pseudomonas sp. IESDJP-V1 (MH362754) and Pseudomonas sp. IESDJP-V2 (MH362755), S. marcescens IESDJP-V3 (MH362756), B. cereus IESDJP-V4 (MH362757), Ochrobactrum sp. IESDJP-V5 (MH362758).

## Declaration of interest’s statement

The authors declare no competing interests.

## Additional information

No additional information is available for this paper.

## Abbreviation

PGPR: Plant Growth-Promoting Rhizobacteria
μL: Micro Liter
BLAST: Basic Local Alignment Search Tool
NCBI: National Center for Biotechnology Information
kg: Kilogram
FYM: Farm yard Manure
CFU: Colony-Forming Unit
mL: Milliliter
mg: Milligrams
g: gram
DNS: Dinitrosalicylic Acid
EC: Electrical Conductivity
μS cm^-1^: millisiemens/centimeter
ANOVA: Analysis of Variance
MR: Methyl red
VP: Voges-Proskauer
IAA: Indole-3-Acetic Acid
HCN: Hydrogen Cyanide
cm: Centimeter
OC: Organic Carbon
OM: Organic matter
CAS: Chrome Azurol S
PCR: Polymerase Chain Reaction
